# Peripheral oxytocin injection modulates vomeronasal sensory activity and reduces pup-directed aggression in male mice

**DOI:** 10.1038/s41598-020-77061-7

**Published:** 2020-11-17

**Authors:** Thiago S. Nakahara, Antonio P. Camargo, Pedro H. M. Magalhães, Mateus A. A. Souza, Pedro G. Ribeiro, Paulo H. Martins-Netto, Vinicius M. A. Carvalho, Juliana José, Fabio Papes

**Affiliations:** 1 Department of Genetics, Evolution, Microbiology and Immunology, Institute of Biology, University of Campinas, Rua Monteiro Lobato, Campinas, SP 13083-862 Brazil; 2 Graduate Program in Genetics and Molecular Biology, Institute of Biology, University of Campinas, Rua Monteiro Lobato, Campinas, SP 13083-862 Brazil

**Keywords:** Olfactory system, Sensory processing, Social behaviour

## Abstract

Behaviors are shaped by hormones, which may act either by changing brain circuits or by modifying sensory detection of relevant cues. Pup-directed behaviors have been previously shown to change via action of hormones at the brain level. Here, we investigated hormonal control of pup-induced activity in the vomeronasal organ, an olfactory sensory structure involved in the detection of non-volatile chemosignals. Vomeronasal activity decreases as males switch from a pup-aggressive state to a non-aggressive parenting state, after they socially contact a female. RNA sequencing, qPCR, and in situ hybridization were used to identify expression, in the vomeronasal sensory epithelium, of candidate GPCR hormone receptors chosen by in silico analyses and educated guesses. After identifying that oxytocin and vasopressin receptors are expressed in the vomeronasal organ, we injected the corresponding hormones in mice and showed that oxytocin administration reduced both pup-induced vomeronasal activity and aggressive behavior. Conversely, injection of an oxytocin receptor antagonist in female-primed male animals, which normally exhibit reduced vomeronasal activity, significantly increased the number of active vomeronasal neurons. These data link oxytocin to the modulation of olfactory sensory activity, providing a possible mechanism for changes in male behavior after social experience with females.

## Introduction

Hormones are key regulators of diverse functions in the body. These substances modulate several animal behaviors, preparing the individual to respond appropriately to the environment, depending on the presence of specific internal and external cues. Hormones have been shown to alter brain activity via action upon cognate receptors in the neural tissue, which in turn lead to behavioral changes. For example, oxytocin, vasopressin, prolactin, and steroid hormones have been extensively investigated and shown to modify brain activity and behaviors as diverse as mating, intermale aggression, parental care, and pup-directed aggression^1–3^.

In particular, oxytocin and vasopressin are able to act at different brain sites to control social behaviors, including social recognition memory, aggressive and competitive behaviors, social bonding, and affiliative pup-oriented behaviors^4–17^. For example, oxytocin modulates nurturing behaviors in mother animals^11^, while vasopressin controls scent marking, aggressive behavior, and offspring care exhibited by fathers^12–17^. These effects seem to be commanded by hormonal action at the brain level, since intracerebral oxytocin injection in pregnant mice increases maternal behavior^18^, whereas administration of oxytocin receptor antagonists impairs maternal behavior^19–21^.

Besides their mode of action at the brain level, hormones may also act directly at the periphery, affecting behaviors by changing the way sensory organs and/or effector tissues function^22^. An example is the action of oxytocin on sweet stimulus detection by taste buds in the tongue, decreasing the display of appetitive behavior^23,24^. Another example involves the influence of progesterone^25^ on the vomeronasal organ (VNO), a sensory structure specialized in the detection of chemosignals that elicit instinctive behaviors^26^ such as male territorial aggression^27^, female sexual receptivity^28^, male sexual behavior^29–31^, and male–female sexual discrimination^32,33^. Progesterone affects the female VNO to reduce detection of male chemosignals, thereby controlling sexual behaviors^25^.

In this paper, we hypothesized that hormones acting directly at the VNO level modulate the organ’s sensory activity, putatively resulting in behavioral changes. We investigated selected GPCR hormone receptor candidates for expression in the VNO and found that some, including oxytocin and vasopressin receptors, are found in this sensory structure. In the context of pup olfactory detection by laboratory mice, we showed that VNO activity and aggression towards pups decreases in the male after social contact with a female^34,35^. Next, we tested the involvement of oxytocin and vasopressin in this phenomenon, finding that treatment with oxytocin, but not vasopressin, was sufficient to decrease activity of VNO sensory neurons and pup-directed aggression in sexually naïve male mice. Importantly, administration of oxytocin receptor antagonist led to an increase in pup-induced VNO activity. Together, these data strongly suggest that vomeronasal neurons sense the individual’s oxytocin levels to change sensory neuron activity. We hypothesize that this phenomenon may mediate changes in pup-driven behaviors.

## Results

### Reduction in pup-directed aggression in male mice is accompanied by changes in vomeronasal neuron activity

**Figure 1:**
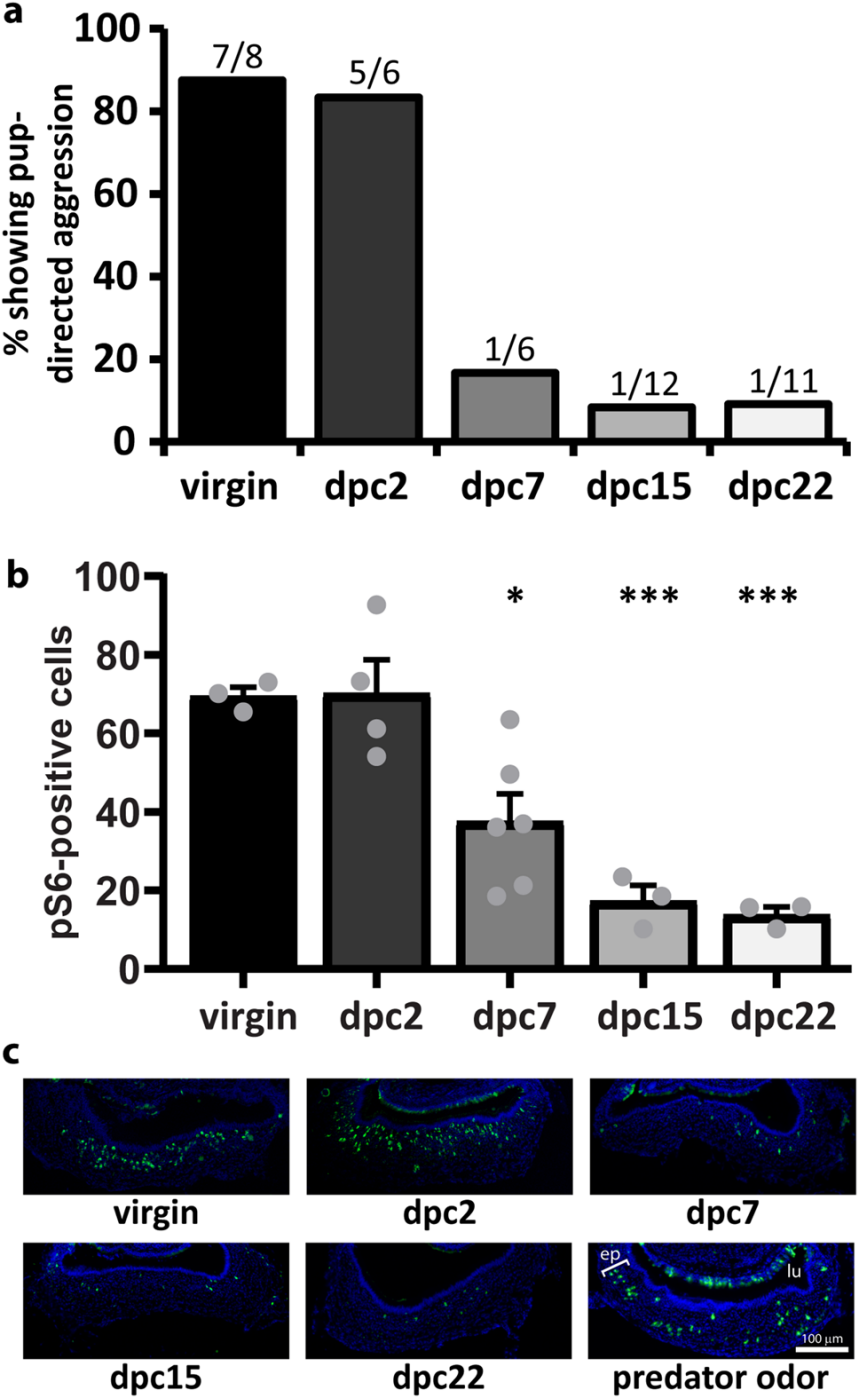
Social experience with a female changes sensory activity and behavior in male mice. (**a**) Percentage of male subjects displaying pup-directed aggression, depending on the duration of cohabitation with a female (dpc). Groups are virgin mice (without contact with a female), dpc2 (2 days of cohabitation), dpc7, dpc15, and dpc22 (n = 6–12 animals). For each group, the number of aggressive individuals out of all mice assayed is represented above the bar. (**b**) Number of vomeronasal neurons immunostained for the activation marker pS6 (normalized to 50,000 μm^2^ of sensory epithelium) in subjects exposed to pup odors in the same groups shown in (**a**) (n = 3–6 animals; mean + S.E.M.; gray dots represent values for individual mice). *P < 0.05; ***P < 0.001; one-way ANOVA followed by Honestly Significantly Different post-hoc analysis; difference relative to ‘virgin’ group. See also Supplementary Table S1 for metrics of statistical tests and means calculated across all sections. (**c**) Representative images of VNO coronal sections subjected to pS6 immunostaining (green fluorescence) in groups shown in (**a**). DAPI nuclear staining is shown in blue. Positive control is VNO from animals exposed to predator odor (cat). *ep* vomeronasal sensory epithelium, *lu* vomeronasal organ lumen. Bar 100 μm.

The main goal of our study was to investigate hormones that directly change activity in the vomeronasal organ of mice. We studied hormonal modulation of VNO activity in the context of pup-directed aggressive behavior in adult males, shown to be mediated by the vomeronasal organ^34–36^. One interesting aspect of pup-directed aggression is that it is exhibited only by virgin (sexually inexperienced) males; after sexual contact with a female, the male undergoes a progressive behavioral switch, culminating in a state in which it no longer displays pup-directed aggression (Fig. 1a)^34,37–41^ and instead exhibits paternal care (retrieval of wandering pups back to the nesting area, pup licking, pup grooming and nesting). This effect seems to occur even when the male is separated from the female and/or pups after a short period of cohabitation^34,35,39,42^, but reverses back to normal aggressive levels after 2 months of social isolation (data not shown)^37^.

We tested pup-directed aggression levels in virgin males and in sexually experienced animals after 2, 7, 15, and 22 days of cohabitation with a female. These groups are hereinafter named virgin, dpc2 (days post cohabitation), dpc7, dpc15, and dpc22, respectively. We observed that the males undergo a behavioral switch after a period of 2 weeks following cohabitation with a female, in which aggression levels are reduced (Fig. 1a), similar to previous observations^34,35,37,39–41^. Importantly, this behavioral change is correlated with striking alterations in VNO activity (Fig. 1b,c; Supplementary Table S1), which starts to decline at dpc7 and reaches the lowest levels at dpc15 and later time points^34,42^. Because sexual interaction between males and females leads to the release of hormones in the male^43^, it is possible that the sensitivity of the male VNO's pup-detecting neurons is decreased by direct hormonal action at the VNO. We hypothesize that this event gates the detection of pup-derived chemical cues, resulting in a decrease in pup-directed aggression.

### A subset of hormone receptors is expressed in the vomeronasal organ

In order to investigate if hormones alter vomeronasal organ activity during the male behavioral switch, we searched for hormone receptors expressed in the VNO. We initially focused on neuropeptide receptors because they include receptors for oxytocin, vasopressin, and prolactin, which have been extensively linked to the modulation of many social behaviors, including pup-directed aggression and infanticide, in a range of animal species^3,43–51^. First, we searched the literature for all known neuropeptide receptors and created an initial driver list with 79 receptors (not shown), whose protein sequences were recovered from the GenBank protein database. We used this list to recover additional annotated or putative receptors with similar amino acid sequences (blastp; cut-off P value < 10^–15^), ending with an expanded list of 126 receptors.

Since a wide variety of neuropeptide receptors belongs to the rhodopsin-like superfamily of G-protein coupled receptors (GPCR)^52^, we searched the PFAM protein database for proteins encoded in the mouse genome that adhered to the *7tm_1* seven-transmembrane domain signature of rhodopsins, retrieving 2182 sequences in total. These sequences cluster into 47 groups (Markov Cluster Algorithm; inflation index = 2.0), three of which contained the 126 neuropeptide receptors retrieved from GenBank and discovered through Blast searches. Together, the 3 identified GPCR clusters contained 435 protein sequences.

Next, transcript isoforms for all 435 proteins were identified in RNA sequencing libraries from several sensory organs, including the two most studied olfactory structures in the mouse nasal cavity, the VNO and the Main Olfactory Epithelium (MOE)^53^ (Supplementary Table S2 contains a comprehensive list of expression levels, in transcripts per million, or TPM, for putative neuropeptide hormone GPCR receptors in the accessory olfactory organs of male and female mice). Expression levels were condensed to the gene level (FPKM), followed by selection of members with FPKM higher than the arbitrary cutoff of 0.5 (Supplementary Table S3), which, in general, are genes more likely to be detected by in situ hybridization in the olfactory organs. We further curated this candidate gene list, excluding from additional analyses those clearly annotated as non-hormone GPCR receptors or linked to biological processes not likely to contribute to olfaction-driven behavior.

**Figure 2:**
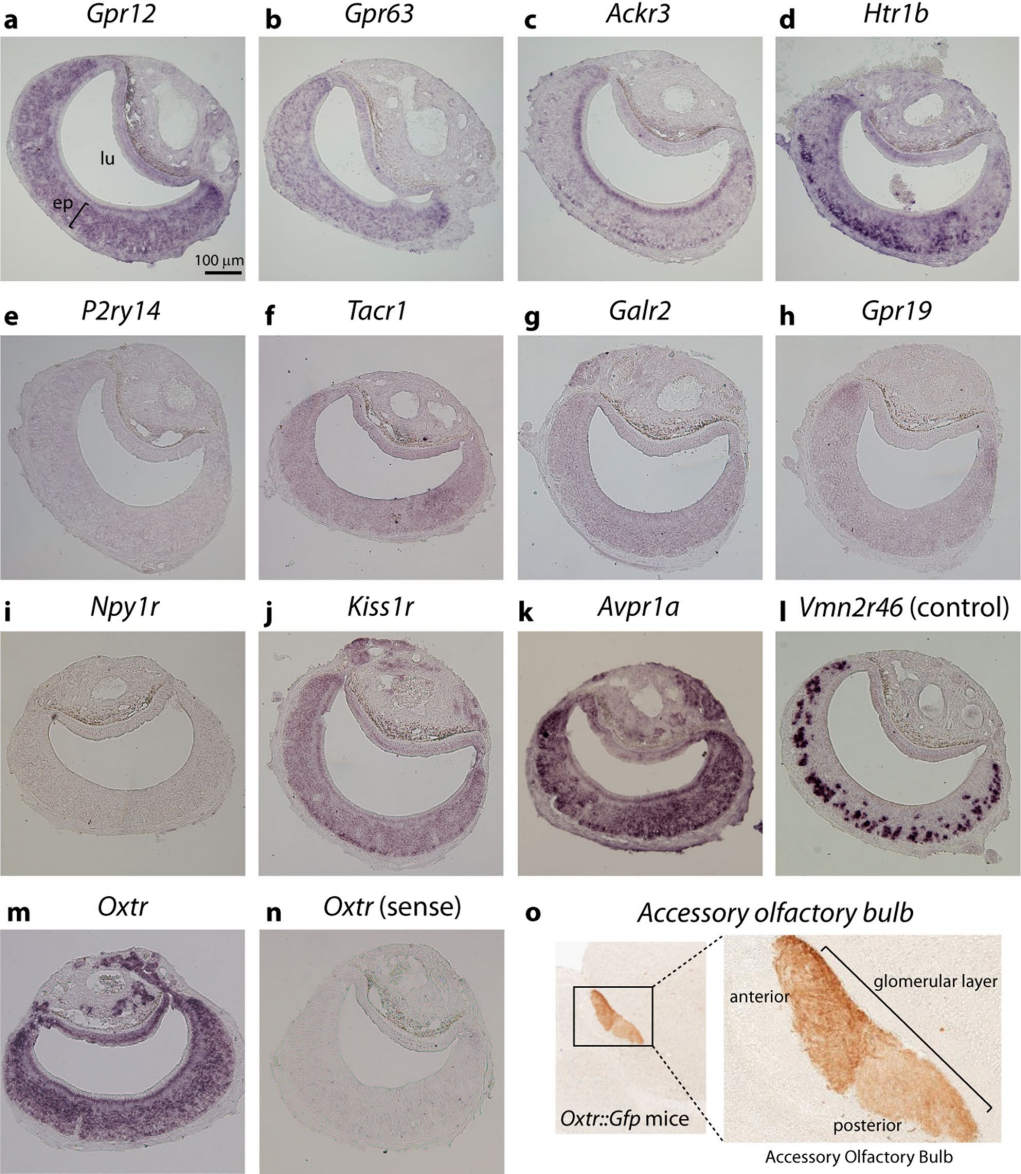
In situ hybridization for candidate hormone receptor genes in the vomeronasal organ of adult virgin male mice. (**a–n**) Each panel shows chromogenic in situ hybridization staining (purple) on VNO coronal sections with probes for genes *Gpr12* (**a**), *Gpr63* (**b**), *Ackr3* (**c**), *Htr1b* (**d**), *P2ry14* (**e**), *Tacr1* (**f**), *Galr2* (**g**), *Gpr19* (**h**), *Npy1r* (**i**), *Kiss1r* (**j**), *Avpr1a* (**k**), and *Oxtr* (**m**). Positive control (**l**) is staining with probe for V2R vomeronasal receptor gene *Vmn2r46*, expected to be expressed in a large set of cells in the VNO's basal zone. For the oxytocin receptor gene *Oxtr*, staining obtained with a control sense probe is shown for comparison (**n**). ep, vomeronasal sensory epithelium; lu, vomeronasal organ lumen. Bar is 100 μm. (**o**) Glomerular layer of the accessory olfactory bulb from *Oxtr::Gfp* transgenic mice where GFP is under the control of the *Oxtr* gene promoter (GENSAT Project database^56^). Immunostaining for GFP is shown in brown. Higher magnification image on the right shows anterior and posterior divisions of the bulb.

This approach resulted in selected putative hormone receptor genes (Table 1; marked in Supplementary Table S3), which were chosen for further confirmation of expression by in situ hybridization on histological sections of VNOs from adult virgin males. Most receptor genes with FPKM values higher than 1.0, such as *Gpr12*, *Gpr63*, *Ackr3*, and *Htr1b*, resulted in unequivocal labeling in the VNO (Fig. 2a–d). *Gpr12* and *Gpr63*, which code for orphan candidate GPCR receptors, were detected across the entire sensory epithelium without any defined spatial pattern (Fig. 2a,b). *Ackr3* weakly stained two populations of cells, one near the base of the epithelium and one in the VNO’s supporting cell layer near the lumen (Fig. 2c). Receptor gene *Htr1b*, which codes for a serotonin receptor subtype, yielded strong in situ hybridization signal in VNO cells near the epithelium’s base (Fig. 2d).

**Table 1:** In silico quantification of expression of selected candidate GPCR genes in RNA sequencing libraries of the vomeronasal organ from C57BL/6 virgin male mice.

Gene	Mean expression	Males (mean ± S.E.M.)	Females (mean ± S.E.M.)	Annotation
*P2ry14*	22.72	22.56 ± 1.49	22.89 ± 2.05	Purynergic receptor P2y, Gpr14
*Ackr3*	12.97	12.88 ± 0.46	13.05 ± 1.55	Atypical chemokine receptor 3 also known as C-X-C chemokine receptor type 7 (CXCR-7)
*Gpr12*	9.58	10.01 ± 0.53	9.15 ± 0.81	G Protein-Coupled Receptor 12
*Htr1b*	2.65	2.42 ± 0.26	2.88 ± 0.24	5-Hydroxytryptamine (Serotonin) Receptor 1B
*Gpr63*	2.14	2.10 ± 0.15	2.18 ± 0.17	G protein-coupled receptor 63
*Tacr1*	1.4	1.15 ± 0.19	1.65 ± 0.54	Tachykinin Receptor 1
*Kiss1r*	0.94	1.05 ± 0.12	0.81 ± 0.08	Receptor for metastin (kisspeptin-52 or kp-52)
*Npy1r*	0.68	0.59 ± 0.10	0.76 ± 0.05	Neuropeptide Y receptor type 1
*Gpr19*	0.63	0.62 ± 0.05	0.65 ± 0.02	G Protein-Coupled Receptor 19

Quantification metric is FPKM (fragments per kilobase per million reads). Gene annotation is shown on the right column. FPKM is shown as mean ± S.E.M (n = 3 mice). For comparison, expression levels in female mice are shown in a separate column (n = 3 mice).

On the other hand, despite its substantial expression levels in VNO RNA-Seq libraries, expression of the *P2ry14* gene could not be detected in the VNO by in situ hybridization, an unexpected result that requires future clarification (Fig. 2e). Probes for *Tacr1*, a gene encoding a tachykinin receptor, did not yield positive hybridization signals in several experiments, probably due to its medium-to-low expression (FPKM ~ 1.0) (Fig. 2f). Most hormone receptor genes with FPKM lower than 1.0 (*Galr2*, *Gpr19*, and *Npy1r*) did not result in positive hybridization staining signal (Fig. 2g–i). Out of all genes with FPKM lower than 1.0, kisspeptin receptor 1 gene (*Kiss1r*) has the highest expression level in VNO RNA-Seq libraries (Table 1); concordantly, we could detect hybridization staining for this gene, which exhibits an indiscriminate spatial pattern throughout the entire sensory epithelium, with a slightly stronger signal at its corners and at foci near the base of the epithelium, where progenitor cells are found (Fig. 2j).

In addition to genes in Table 1, we also performed in situ hybridization with probes for oxytocin receptor gene *Oxtr* and vasopressin receptor genes *Avpr1a*, *Avpr1b* and *Apvr2*, which were chosen as educated guesses, due to their involvement in the control of social behaviors in mice^16,49,54,55^. *Avpr1b* and *Apvr2* were not found in the male VNO (not shown), but we observed strong signals for one subtype of vasopressin receptor, *Avpr1a* (Fig. 2k), and for *Oxtr* (Fig. 2m; positive control gene *Vmn2r46* is shown in Fig. 2l). In both cases, probes stained cells across the entire sensory epithelium, without any discernible preferential spatial pattern (Fig. 2m,k; control sense probe in Fig. 2n), and the staining developed fast, suggesting robust expression levels in the VNO. We also observed the presence of GFP-labeled axons in the glomerular layer of the accessory olfactory bulb in *Oxtr::GFP* transgenic mice (Gene Expression Nervous System Atlas—GENSAT Project database^56^; Fig. 2o). These labeled fibers exhibit a spatial pattern similar to the innervation of the glomerular layer of the accessory olfactory bulb by VNO sensory neuron axons, and therefore these data further suggest that *Oxtr* is indeed expressed in VNO neurons, although we cannot rule out the alternative possibility that the GFP-labeled axons come from other brain regions.

Together, these data show expression of some GPCR hormone receptors in the VNO, including oxytocin receptor *Oxtr* and vasopressin receptor *Avpr1a*.

### Oxytocin and vasopressin receptors are expressed throughout the VNO epithelium in male mice

Because we wanted to find hormones that act to modulate VNO activity in response to pups, we screened hormone receptors investigated in Fig. 2 and selected those expressed in VNO cells activated by pup odors during the display of pup-directed aggression. The VNO epithelium is highly activated by exposure to pup odors (see Methods for details on olfactory stimulation), as judged by the expression of phosphorylated ribosomal protein S6 (pS6) (Fig. 1b), a surrogate marker of vomeronasal neuron activity^57–59^. Although active cells are located throughout the VNO epithelium in virgin males (Fig. 1c), they seem to be preferentially located in the apical zone (Supplementary Fig. S1), in accordance with published data showing that pup-activated cells are apically located in the neuroepithelium and that genetic ablation of apical neurons severely impairs pup-directed aggression^34,60^.

Out of all receptors expressed in the vomeronasal epithelium (Fig. 2), *Kiss1r*, *Ackr3*, *Htr1b* are preferentially expressed in the basal zone and so were discarded as candidate hormone receptors potentially involved in the control of pup-directed aggressive behavior in males. *Gpr12* and *Gpr63* are suspected to be sphingosine-1-phosphate receptors, not hormone receptors, and therefore their possible roles in VNO modulation was not pursued further^61–63^.

On the other hand, *Oxtr* and *Avpr1a* hybridization stainings were found to be expressed throughout the VNO epithelium, not exclusively in the basal zone (Fig. 2k,m,n) and therefore were chosen as candidates hormone receptors involved in the modulation of pup-detecting VNO cells.

**Figure 3:**
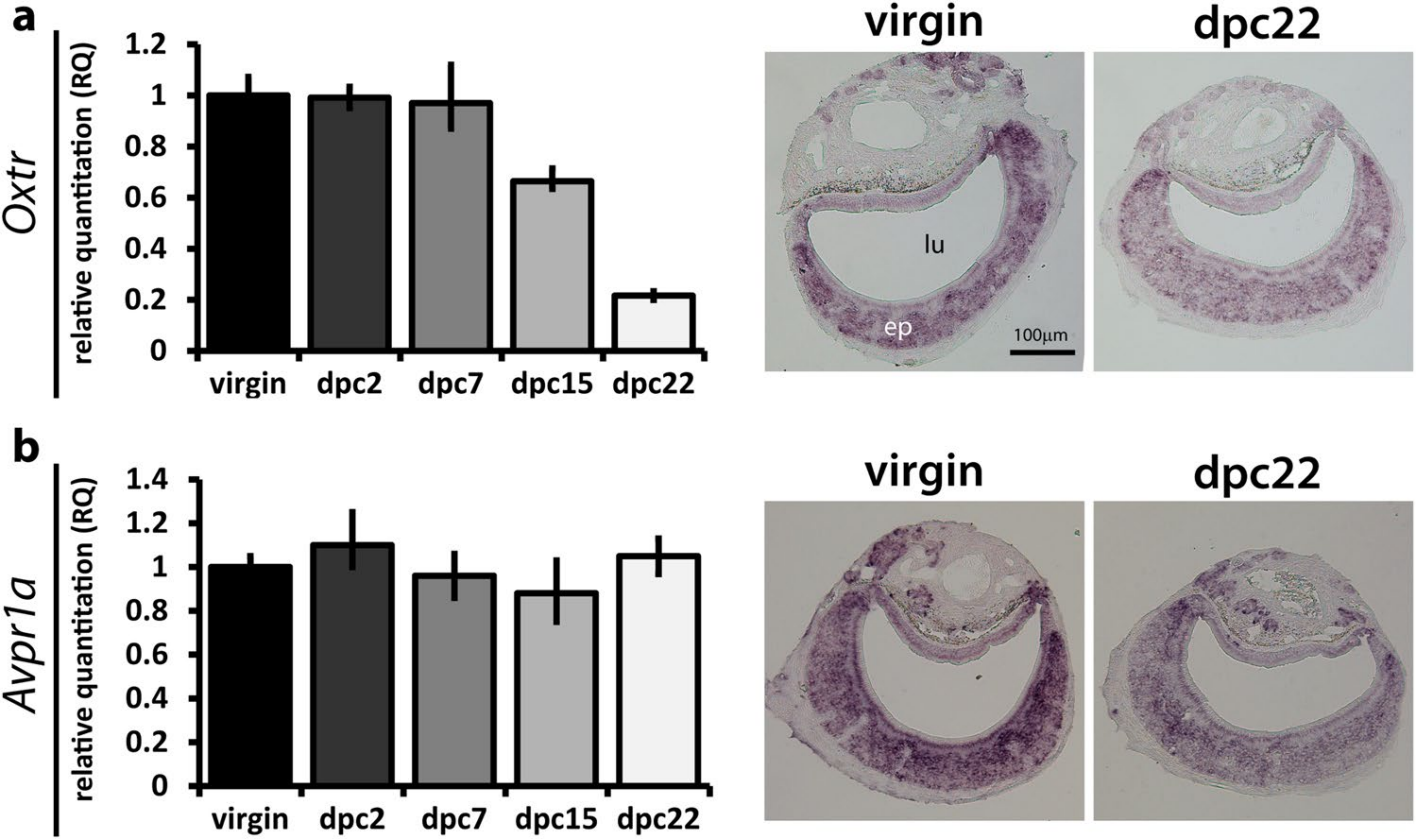
Confirmation of expression of oxytocin receptor gene *Oxtr* and vasopressin receptor gene *Avpr1a* in the VNO by quantitative PCR and in situ hybridization. Left panels: relative expression quantitation (RQ) determined by RT-qPCR with TaqMan probes for *Oxtr* (**a**) and *Avpr1a* (**b**) in the VNO (n = cDNAs from 3 animals (biological replicates) per group, 3 technical replicates per biological replicate). RQ values are relative to mean expression level of the ‘virgin’ male group. Right panels: representative images of in situ hybridization experiments for *Oxtr* (**a**) and *Avpr1a* (**b**) on VNO coronal sections from virgin and dpc22 animals. *ep* vomeronasal sensory epithelium, *lu* vomeronasal organ lumen. Bar is 100 μm.

Next, we investigated *Oxtr* and *Avpr1a* expression in the VNO by reverse transcription-coupled qPCR. Our hypothesis for this study is that a hormone would act upon its cognate receptor(s) in the VNO at the time the virgin male socially experiences the female, producing consequential changes in sensory activity and behavior. One requirement for this hypothesis is that *Oxtr* needs to be expressed in virgin males and/or during the first days of cohabitation, that is, at any point during the window from virgin to dpc7, which would allow oxytocin to change sensory activity and aggressive behavior (Fig. 1). We used RT-qPCR to test this hypothesis, using samples from virgin male mice and dpc2, dpc7, dpc15, and dpc22 time points after the onset of cohabitation (Fig. 3a). *Oxtr* expression was found in virgin males up until dpc7, starting to decline at dpc15 and reaching the lowest levels in parenting males (dpc22) (Fig. 3a, left). These data prove that expression of *Oxtr* occurs during the period in which VNO activity and pup-directed behavior change (Fig. 1). Similarly, *Avpr1a* is also expressed in the VNO during the virgin-to-dpc7 time window (Fig. 3b, left).

We also verified the pattern of expression of *Oxtr* and *Avpr1a* in the VNO by in situ hybridization in the virgin and dpc22 groups of male mice. Labeling with an *Oxtr* probe is very evident in both groups, with strong staining across the VNO sensory epithelium in virgin males and weaker staining in dpc22 males, in accordance with the lower expression in this group detected by qPCR (Fig. 3a, right). Staining for *Avpr1a* is found throughout the epithelium in virgin and in dpc22 mice (Fig. 3b, right).

Together, our in situ labeling and qPCR data confirm expression of *Oxtr* and *Avpr1a* receptors in the VNO, and show that they are present in the virgin-to-dpc7 time window, after which VNO activity and behavior start to change.

### Oxytocin modulates activity of vomeronasal neurons

Oxytocin and vasopressin both act in the brain to change neural activity and social behaviors^2,48,49,54,55,64^. Our hypothesis is that these hormones also act directly at the sensory interface to change VNO activity towards pup odors and behavior. One prediction from this model is that administration of such hormones would alter pup-induced VNO activity.

**Figure 4:**
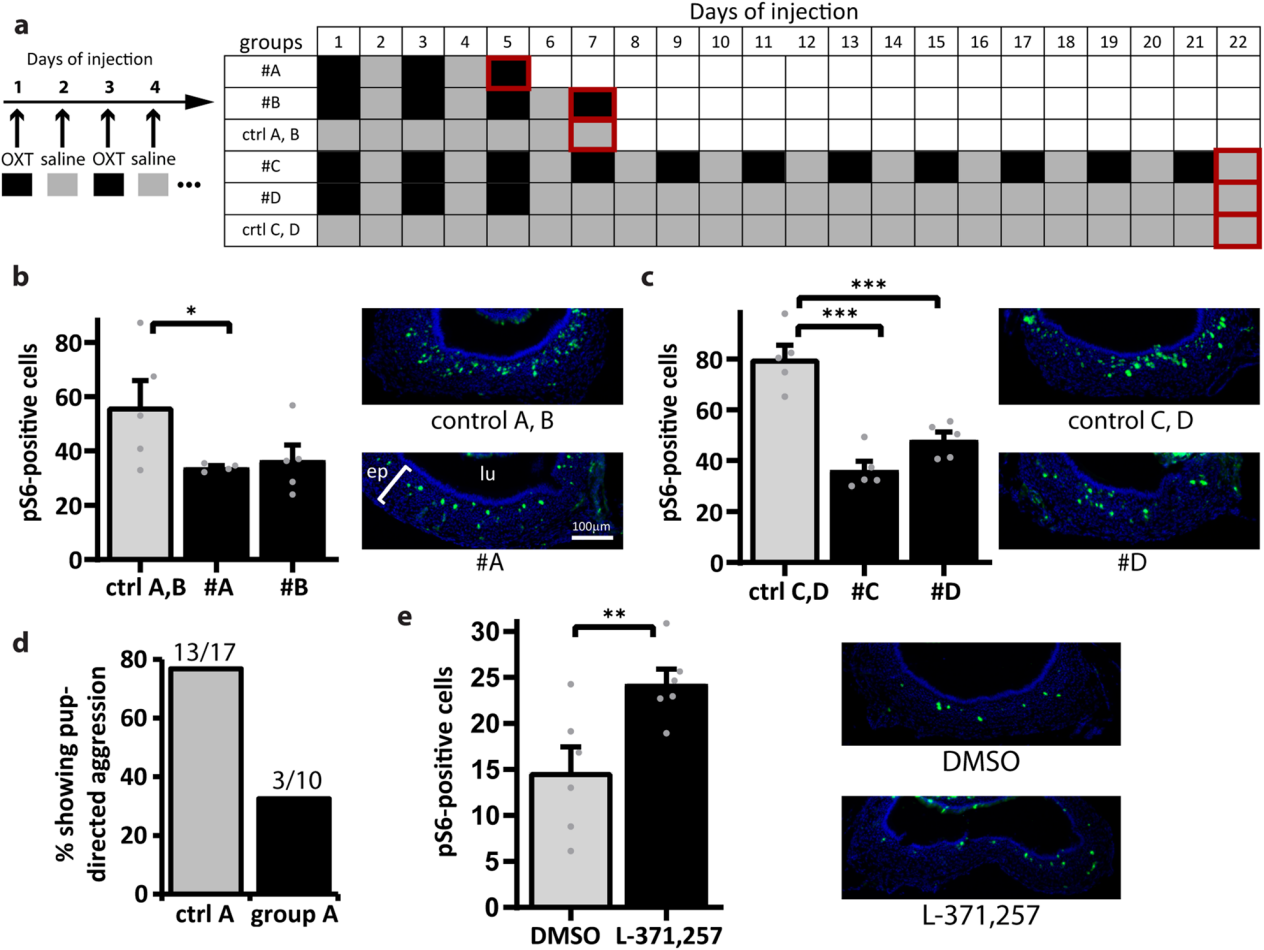
Manipulation of oxytocin function alters VNO activity in male mice exposed to pup odors. (**a**) Regimens of oxytocin (agonist) intraperitoneal injections in adult virgin mice. *OXT* oxytocin. Numbers represent days since the first injection. Black cells (right) represent days with oxytocin injection; gray cells indicate days with saline injection. Groups #A and #B represent situations where oxytocin is injected for a few days and the animals are analyzed immediately after. Group #C includes animals subjected to long-term oxytocin injection for 21 days. Group #D has mice injected with oxytocin for a few days followed by several days without oxytocin injection prior to analysis. Cells with red outlines are days when injected virgin animals were exposed to pup odors, evaluated for pup-directed aggressive behavior and euthanized to harvest VNOs for activity assays. (**b**) Number of active VNO cells (normalized to 50,000 μm^2^ of sensory epithelium), as judged by pS6 immunostaining (green fluorescence in representative VNO coronal section images on the right), after exposure of injected virgin animals in groups #A or #B to pup odors. DAPI nuclear stain is shown in blue. n = 4–5 animals; 3–6 sections per animal; gray dots represent values for individual mice. See Supplementary Table S1 for statistical test metrics and means across all sections. (**c**) Similar to (**b**), but for injected virgin animals in groups #C and #D, according to regimens in panel (**a**). n = 5 animals; 3–6 sections per animal. (**d**) Percentage of subjects displaying pup-directed aggression in control (n = 17 mice) and oxytocin-injected (n = 10 mice) virgin animals (group #A). For each group, the number of aggressive individuals out of all mice assayed is represented above the bar. (**e**) Left panel: number of pS6-positive cells in the VNO (normalized to 50,000 μm^2^ of sensory epithelium) from dpc15 animals injected with oxytocin receptor antagonist L-371,257 (n = 6 mice) or DMSO (control; n = 6 mice). Right panel, corresponding representative images of VNO coronal sections subjected to pS6 staining. *Ep* VNO epithelium, *lu* VNO lumen. *P < 0.05, **P < 0.01, ***P < 0.001; one-tailed Welch's t test assuming unequal variances. Bar is 100 μm.

It is extremely challenging to measure plasma concentrations of oxytocin and vasopressin, due to their labile nature; even though an increase in plasma oxytocin has been described in males after sexual experience with the female^43^, the exact temporal profile of the hormone surge in the plasma is unknown. In our experiments, we decided to inject oxytocin and vasopressin in virgin male mice using hormone administration regimens of distinct temporality. In group A, animals were subjected to three intraperitoneal oxytocin injections on alternate days and analyzed on day 5 (Fig. 4a). In group B, animals were injected four times on alternate days and analyzed on day 7 (Fig. 4a). Because pup-directed aggression and VNO activity decrease sharply between dpc2 and dpc7 (Fig. 1a), groups A and B simulate an acute increase in oxytocin during that period. On the other hand, we created experimental groups in which the injections in virgin male mice were prolonged: group C is characterized by oxytocin administration every other day for 22 days (Fig. 4a), while group D included mice injected every other day for 5 days at the beginning of the treatment regimen, followed by a period without injections (Fig. 4a), to simulate a surge in hormone concentration.

In groups A and B, a marked reduction in pup-induced vomeronasal activity was observed (Fig. 4b; see also Supplementary Table S1), indicating that a few days of increased oxytocin are sufficient to modulate the sensory interface activity, phenocopying the decrease in the number of active VNO cells naturally observed at dpc7 (Fig. 1b). A similar reduction was seen in groups C and D (Fig. 4c; Supplementary Table S1). Interestingly, it seems that the effects of oxytocin on VNO activity remain for several days after withdrawal of hormone injections (group D), suggesting that a hormone surge is enough to modulate VNO activity and change the ensuing behavior accordingly (Fig. 4c,d). When an injection regimen similar to group A was performed with vasopressin, VNO activity increased (Supplementary Fig. S2; Supplementary Table S1), which is in contrast to the decrease observed during the behavioral transition from aggression to lack of aggression in unmanipulated males (Fig. 1).

Strikingly, oxytocin injection led to marked reduction in the percentage of pup-oriented aggressive males (Fig. 4d). Together, these results indicate that oxytocin, but not vasopressin, is able to modulate VNO activity concomitant with changes in behavior, suggesting that this hormone modulates VNO sensory activity and may control pup-driven aggression in males.

### Manipulation of oxytocin signaling changes VNO activity

The injection experiments described in the previous section allowed us to conclude that oxytocin changes VNO activity when injected intraperitoneally. However, these manipulative experiments do not definitively link reduction in VNO activity to oxytocin’s action at the VNO level, because the hormone may additionally act at the brain level in injected mice. Therefore, we adopted a complementary strategy in which an oxytocin receptor antagonist, L-371,257, was intraperitoneally administered to male animals. We chose this specific antagonist because it is unable to cross the blood–brain barrier^65^, and therefore its consequences on VNO activity could be unequivocally assigned to direct manipulation of the sensory interface.

We injected L-371,257 in dpc15 mice, which exhibit naturally reduced pup-induced VNO activity after the male’s contact with the female. Strikingly, the number of pS6-positive vomeronasal cells increased significantly after a single dose of the antagonist (Fig. 4e; Supplementary Table S1), suggesting that oxytocin modulates VNO activity by acting directly at the sensory epithelium level.

Together, the oxytocin and oxytocin receptor antagonist experiments strongly support the hypothesis that this hormone is mechanistically involved in regulating VNO activity levels, acting during the transition from a highly active VNO in aggressive virgin males to an almost inactive VNO in non-aggressive males after contact with female.

### Social contact with female changes male VNO activity in response to predator chemosignals

As outlined in the Introduction, the vomeronasal organ mediates a range of instinctive behaviors linked to the detection of several kinds of chemosignals, including intraspecies and interspecies communication signals^66^. Because oxytocin receptor and other GPCR receptors are found throughout the vomeronasal epithelium, not just in cells activated by pup odors (Figs. 2, 3), one prediction is that vomeronasal activity changes in the male after social interaction with the female not only in the context of pup-derived pheromones but also towards other VNO stimuli.

**Figure 5:**
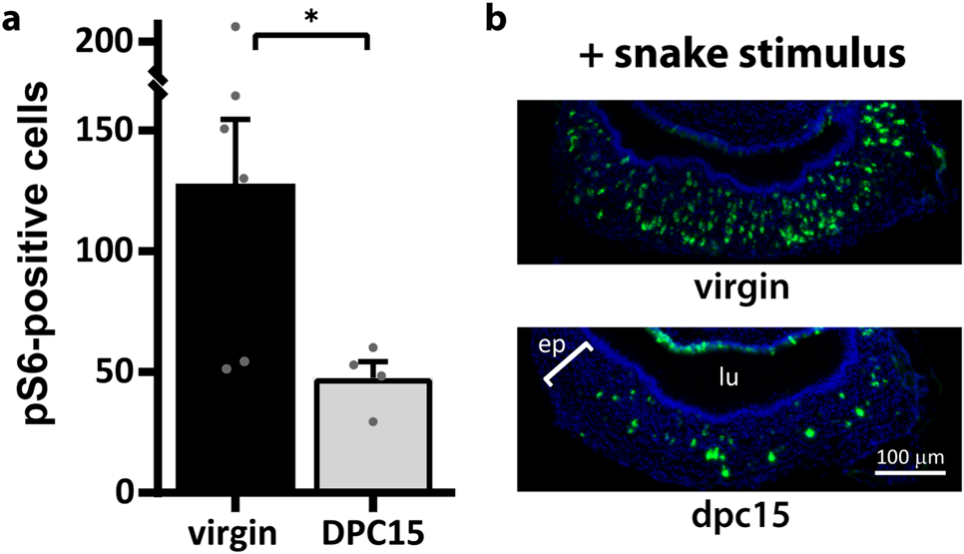
Social experience with a female changes sensory activity in response to predator odor in male mice. Left: number of pS6-positive neuron counts in the VNO (normalized to 50,000 μm^2^ of sensory epithelium), in virgin male mice and in males after 15 days of contact with a female (dpc15) exposed to snake stimuli (n = 4–6 animals; 6–13 sections per animal; gray dots represent values for individual mice). *P < 0.05, one-tailed Welch's t test assuming unequal variances. See also Supplementary Table S1 for statistical test metrics. Right: representative microscopy images of VNO coronal sections after pS6 immunostaining (green fluorescence) from subjects in (**a**). DAPI nuclear staining is shown in blue. *ep* VNO epithelium, *lu* VNO lumen. Bar is 100 μm.

To test this hypothesis, we used predator-derived odors, which elicit VNO activity more strongly than conspecific chemosignals in non-manipulated animals, in both the apical and basal layers of the sensory epithelium^29,66,67^. Snake odors, chemosignals that trigger instinctive defensive avoidance behavior in male mice^66,68^, produced robust VNO sensory neuron activation in virgin individuals (Fig. 5). In contrast, snake-induced VNO activity was significantly reduced in dpc15 males (Fig. 5; Supplementary Table S1), similar to the reduction seen in the context of pup-directed aggression (Fig. 1b), suggesting that the internal hormonal state is able to also modulate sensory activity to predator odors and potentially to several other VNO stimuli.

Even though further experiments are needed to mechanistically link oxytocin or other hormones to the modulation of VNO activity toward stimuli other than pup odors, our data suggest that changes in hormonal state after the male is placed in contact with a female underlie alterations in vomeronasal organ sensory activity.

## Discussion

Our central hypothesis in this study was that a salient social event leads to changes in internal state and hormonal levels, which in turn modify the sensory organ sensitivity to environmental stimuli, possibly influencing the display of behaviors. To test this hypothesis, we chose an experimental paradigm in which there is a change in sensory activity (Fig. 1b) after a social event (contact with the female), concomitant with changes in behavior (pup-directed aggression) (Fig. 1a). We screened a large number of GPCR receptors that may function as hormone receptors, looking for the expression of these candidates in an olfactory organ known to be involved in the control of pup-driven behavior, the vomeronasal organ^34,35,42,69^ (Table 1 and Supplementary Tables S2, S3). Most of these putative receptors showed absent or low expression in the VNO according to RNA sequencing library expression data (Table 1) and in situ hybridization (Fig. 2). Of those found in the VNO by in situ labeling, some exhibited preferential spatial expression in the basal VNO zone (Fig. 2), which does not harbor most of the pup-responsive cells.

In contrast, oxytocin receptor *Oxtr* and vasopressin receptor *Avpr1a* genes were found to be expressed throughout the vomeronasal epithelium (Fig. 2) and were shown to be present at the virgin-to-dpc7 time window (Fig. 3) that precedes changes in VNO activity after the male’s contact with the female. Further investigation of the cognate hormones, oxytocin and vasopressin, showed that administration of oxytocin in virgin male mice led to a decrease in the number of VNO cells activated by pup odors (Fig. 4b,c) as well as in pup-directed aggression (Fig. 4d). Additionally, injection of oxytocin receptor antagonist in males with low VNO activity (non-aggressive dpc15 males) resulted in an increase in VNO activity (Fig. 4e). Together, our data suggest a mechanistic link between oxytocin and the control of vomeronasal activity in male mice. Moreover, we concluded that the changes in VNO activity in males are not restricted to pup-derived stimuli, since this phenomenon also applies to animals exposed to other VNO chemosignals, such as predator odors (Fig. 5).

The implications of our findings are manifold. A recent publication described that hormonal changes during the estrus cycle in female mice control neural activity in a subset of VNO sensory cells able to detect male chemosignals^25^. It is unclear if this is an isolated instance of direct hormonal control of detection of biologically relevant stimuli by the sensory interface, or whether it will prove to be a more general phenomenon. Part of this uncertainty stems from the fact that hormone receptors have not been investigated in detail in the olfactory sensory organs.

We have now screened for the expression of a wide range of candidate GPCR hormone receptor genes in the VNO and found that some, including kisspeptin receptor *Kiss1r*, serotonin receptor *Htr1b*, along with other chemokine receptors, are expressed in the VNO. In future studies, it will be interesting to evaluate which sensory stimuli activate the VNO neurons expressing serotonin receptors, and how and in which biological circumstances the cognate hormone changes sensory activity and behavior.

Importantly, oxytocin receptor gene *Oxtr* was found to be widely expressed in this sensory structure, and we obtained evidence that it controls activity towards several vomeronasal chemosignals. It is important to make it explicit that the finding of oxytocin receptor in the VNO and that its cognate hormone acts to alter sensory organ activity does not preclude its concomitant action at circuits in the brain^35,70^.

Hormonal control of the sensory interface may provide a modulation mechanism to change how the olfactory organs perceive the external world depending on the organism’s internal state, in addition to long-term changes in the repertoire of expressed olfactory receptors in response to the set of chemosignals present in the environment^71^. It may be biologically beneficial to have a system to directly control the sensory interface, preventing the gating of ethologically irrelevant sensory information and of salient stimuli to which the animal should not respond in a particular social situation.

*Oxtr* expression is high in virgin males and during the first days of cohabitation with a female (Fig. 3a). In turn, VNO activity and pup-driven behavior start declining at some point between 2 and 7 days of cohabitation. Our data do not provide a causal link between oxytocin-mediated changes in VNO activity and oxytocin-led changes in behavior. Further experiments are needed to establish causality, but, if this is proven to be the case, how would the oxytocin receptor lead to changes in VNO activity and behavior after a few days of social interaction between the male and its female partner? We would like to put forth two models, which could be objectively tested in future studies. First, it is possible to envision the scenario where oxytocin acts upon its cognate receptor during the first days of contact with a female, after which time the VNO sensory neurons undergo long-term changes in transcription of target genes, modifying their sensitivity to stimuli and, consequently, behavior after a certain number of days (~ 7 days). In another scenario, oxytocin acts on its receptor to promote changes in the proportions of the many distinct types of VNO sensory neurons (each characterized by the expression of a unique olfactory receptor). Through the VNO’s natural turnover process^72^, the newly formed neurons would compose an epithelium with a distinct repertoire of sensory neurons, in turn changing the VNO’s sensitivity to stimuli.

This study focused on oxytocin and its involvement in the detection of pup- or predator-derived odors, and further studies are necessary to investigate whether this hormone also acts in other olfactory organs in the nasal cavity, such as the main olfactory epithelium, mainly involved in the detection of odorants^72^. Our study provides strong support to the hypothesis that oxytocin acts via its cognate receptor at the VNO level to directly change sensory neuron activity. Unfortunately, it was not possible to investigate the consequences of antagonist treatment on behavior, because the injections were made in the presence of 5% DMSO (L-371,257 vehicle), which may have caused some level of behavioral alteration per se, such as general malaise, hampering the interpretation of behavior in our pup-oriented paradigm. Further experiments using *Oxtr* VNO-conditionally knockout mice or injection of oxytocin receptor antagonist followed by behavioral assays are needed to confirm that the oxytocin-led alterations in VNO activity are causally related to changes in the display of pup-directed aggression that occur after the male’s contact with the female.

## Methods

### Animals

Animals were 2- to 4-month-old male C57BL/6 mice, unless otherwise noted. Juveniles used for VNO activation were P0.5 to P8.5 C57BL/6 pups. Subjects used in this study were obtained directly from our vivarium facility.

### Behavioral analysis

Virgin subjects were individually caged for a period of at least 4 days before the onset of the experiment. For the cohabitation experiments, individually housed males were paired with one female per subject, for the duration indicated in each experiment, and transferred to a new cage without the female for 3 h prior to olfactory exposure experiments. pS6 expression and behaviors were assayed in the same individuals, without re-utilization of subjects. Adult subjects were exposed to one or two C57BL/6 pups placed away from the nesting area. Behavioral sessions ran for a maximum period of 15 min, during which time the adult mouse was allowed to freely interact with the pup(s), except when a pup was aggressed upon, in which case this phase of the assay was terminated. Behavior scoring was performed simultaneously by two experimenters, who were kept blind to the subjects’ groups. Pup-directed aggression episodes were counted based on the following criteria: (a) frequent pup squeaking (less than 2 s apart), which is very different from occasional adult grooming-induced pup squeaking (more than 2 s apart); (b) visual inspection that the pup had suffered attack and been wounded; (c) concordant behavior calling by both experimenters. When the VNOs would be subsequently analyzed via pS6 immunostaining, the initial 15 min period was followed by an additional 45 min (total exposure = 60 min), during which time the adult was allowed to interact with 1–3 pups placed inside a plastic capsule containing holes big enough to allow the adult male to poke its nose inside and sniff at the pups but not exhibit aggressive bouts towards them. Immediately after olfactory exposure, subjects were euthanized, followed by VNO dissection and processing for pS6 immunostaining.

### RNA-Seq data analysis

RNA-Seq data from adult VNOs were retrieved from the SRA database (ERR036348, ERR036349, ERR036350, ERR036351, ERR036352, ERR036353)^53^. Reads were mapped to the GRCm38 genome using STAR (version 2.3) and the number of reads aligned to each gene of the Ensembl mouse genome annotation (release 68)^73^ was retrieved with the HTSeq package (version 0.5.4), using the *intersection-nonempty* mode of the *htseq-count* command. Representative FPKM values for each gene were obtained by taking the mean abundance of each gene across the six VNO libraries.

### Tissue preparation

Dissected VNOs were incubated in 30% sucrose/0.45 M EDTA/1 × PBS solution for 5 min, followed by micro-dissection to remove the thicker septal bones, and flash freezing under OCT freezing medium (Leica) without fixation (as needed for subsequent pS6 immunostaining). Cryo-sectioning was performed on a Leica CM1850 cryostat to obtain 16 μm sections, which were stored at − 80 °C. All VNO sections used in this paper were produced by positioning the organ’s long axis perpendicular to the cryostat’s cutting plane. This orientation is sometimes referred to as “transversal”, but can also be approximated to the coronal orientation, because the VNO is a tubular organ whose long axis runs inside the nasal septum approximately in an anterior–posterior orientation.

### Immunostaining

Microscope slides were dried using a blow-dryer for 5 min and fixed with 4% PFA/1 × PBS for 15 min. Sections were permeabilized with 0.1%Triton X-100/1 × PBS for 5 min and washed twice with 1 × PBS, 5 min each. Sections were then blocked for 1 h using Blocking Buffer (1% Blocking Reagent, Tyramide Superboost; Thermo Fisher) at room temperature, followed by a second blocking with 0.1%Triton X-100/1% BSA/1 × PBS for 30 min. Slides wee incubated in a solution containing rabbit anti-pS6 antibody (1:200; Invitrogen Phospho-Ribosomal Protein S6 pSer244/pSer247 Cat. Num. 44-923G) in 0.1%Triton X-100/1% BSA/1 × PBS overnight at 4 °C. Slides were washed 3 times for 5 min in 0.1% Triton X-100/1 × PBS and incubated with anti-rabbit HRP-conjugated antibody (1:100; Tyramide Superboost, Thermo Fisher) for 90 min at room temperature. After 3 additional washes in 0.1% Triton X-100/1 × PBS, Tyramide-Alexa 546 labeling reaction was performed for 7 min (1:100 in 0.0015% H_2_O_2_). After final washes in 1 × PBS (5 min each), slides were counterstained with Hoechst 33,258 (15 μg/ml in 1 × PBS) for 30 min and mounted using 1% Mowiol medium. For measuring the number of pS6-positive cells in the VNO, we counted the number of stained cells in each imaged section and used ImageJ to measure the area of the entire sensory epithelium in that section. The total area was similar across sections and mice (~ 50,000 μm^2^), but we decided to present the number of pS6-positive cells normalized to 50,000 μm^2^ in graphs in Figs. 1, 4, 5, and Supplementary Fig. S1. Images from *Oxtr::Gfp* transgenic mice were obtained from the Gene Expression Nervous System Atlas (GENSAT) Project, NINDS Contracts N01NS02331 and HHSN271200723701C to The Rockefeller University (New York, NY, USA).

### *qPCR for* Oxtr* and* Apvr1a

Three animals from each of the five groups (virgin, dpc2, dpc7, dpc15 and dpc22) were dissected to obtain the VNOs and RNA was extracted with Trizol reagent (Invitrogen). cDNA synthesis was performed using Superscript II reverse transcriptase kit (Thermo Scientific). Briefly, 5 µg total RNA were incubated with 500 ng oligo-dT_12-18_, 1 µl 10 mM dNTPs at 65 °C for 5 min and immediately chilled on ice. The following components were added sequentially to the reaction tube: 4 µl 5 × First Strand Buffer, 2 µl 0.1 M DTT e 1 µl 40 u/µl RNAseOUT, and 0.5 µl of Superscript II (100 u). We performed reverse transcription at 42 °C for 2 h, followed by enzyme inactivation at 70 °C for 15 min. TaqMan qPCR reaction assays (Thermo) were used for *Oxtr* and *Avpr1a* genes, and endogenous control genes were *Actb* (β-actin) and *Gapdh,* with the following cycling parameters on a StepOne equipment (Applied Biosystems): 95 °C for 2 min, 40 cycles of 95 °C for 15 s and 60 °C for 1 min. Relative gene expression quantitation was obtained using the ∆∆Ct method.

### In situ hybridization

For all experiments, we used DIG-labelled complementary RNA probes produced by in vitro transcription with DIG rUTP in a reaction containing 1 kb fragments of the corresponding gene in pGEM-T-Easy vector (Promega). We used SP6 or T7 RNA polymerase (Roche), depending on the orientation of the fragment and the desired probe (sense or antisense). Sixteen micrometer cryostat sections were fixed in 4% PFA/1 × PBS solution for 20–30 min, followed by acetylation with acetic anhydride in 0.1 M triethanolamine (pH 8.0) for 10 min. All incubations were preceded by two washes in 1 × PBS. For the hybridization step, we used 1500 ng/ml DIG-labeled probes in a solution containing 50% formamide, 10% dextran sulfate, 600 mM NaCl, 200 μg/ml yeast tRNA, 0.25% SDS, 10 mM Tris–HCl pH 8.0, 1 × Denhardt’s solution, and 1 mM EDTA pH 8.0. Following hybridization at 60 °C or 62 °C for 16 h, slides were washed in 2 × and 0.2 × SSC (30 min each), ending with a 0.1 × SSC wash for 20 min. All washes were conducted at 55 °C. After washes, immunostaining to detect DIG was performed by blocking in 100 mM Tris–HCl pH 7.5, 150 mM NaCl, 0.05% blocking reagent (Perkin Elmer), and incubation with anti-DIG alkaline phosphatase-conjugated antibody (Roche) at 4 °C (1:800 in the same buffer). BCIP/NBT (Sigma) signal development was performed at room temperature for 16 h.

### Oxytocin and oxytocin receptor antagonist administration

Adult virgin male mice were injected i.p. with oxytocin (68 μg/kg of body weight; Sigma). Injection regimens were offered every other day, alternated with i.p. saline injections. Individuals in the control group were injected daily with 85 μl of saline. Oxytocin doses were decided based on similar regimens used in the literature shown to drive significant increases in oxytocin plasma concentrations without causing malaise^24,74,75^. In experiments where VNO activity was assayed, animal stimulation was performed for 1 h with pup odors, following the methods described above. For oxytocin receptor antagonist treatment, dpc15 males (15 days of cohabitation with a female) were injected with a single dose of oxytocin receptor antagonist L-371,257 (10 mg/kg; Tocris). Initially, the antagonist was dissolved in pure DMSO, to ensure complete solubilization, and then the appropriate dose (~ 50 μl) was diluted in 1 ml saline (final concentration of DMSO is 5%). Control groups were injected with vehicle (5% DMSO) only. It was not possible to assess changes in behavior in animals injected with the antagonist because we detected an increase in the percentage of pup-directed aggression in both antagonist and control groups. VNOs were dissected and processed to evaluate activation levels via pS6 immunostaining at least 2 h after oxytocin antagonist injection. For vasopressin treatment, virgin male mice were injected i.p. with vasopressin (400 μg/kg of body weight; Tocris)^24^, using an injection regimen with 3 doses offered on alternate days, similar to group A in Fig. 4a.

### Statistical analyses

Statistical analyses were performed as previously published^42^, using GraphPad Prism, *R* package, and XLSTAT add-on for Excel. One-way ANOVA and Tukey–Kramer HSD post-hoc test was performed for comparing mean cell counts and behavioral outputs. For comparing mean cell counts in oxytocin, vasopressin and oxytocin receptor antagonist treatments, we used two-sample Welch’s t test considering unequal variances. *P* value is the probability that the null hypothesis (means are equal) is true, and *P* less than 0.05 led to null hypothesis rejection. See Supplementary Table S1 for details on statistical tests and metrics.

## Ethical approval

Animal procedures were carried out in accordance with Animal Protocol no. 1883-1, approved by the Institute of Biology’s Institutional Animal Care and Use Committee (Committee for Ethics in Animal Use in Research), at the University of Campinas. This protocol follows the guidelines established by the National Council for Animal Experimentation Control (CONCEA-Brazil).

## Supplementary information

Supplementary Information.

Supplementary Table S2.

Supplementary Table S2.

Supplementary Table S3.
