# Supplementary material for: Peripheral oxytocin injection modulates vomeronasal sensory activity and reduces pup-directed aggression in male mice
Source: Sci Rep. 2020 Nov 17;10:19943. doi: 10.1038/s41598-020-77061-7 (PMC7673031; doi:10.1038/s41598-020-77061-7)
Supplement: Supplementary file 1 — Supplementary Information. [file 41598_2020_77061_MOESM1_ESM.pdf]

Supplementary Information

Peripheral oxytocin injection modulates vomeronasal sensory activity and reduces pup-directed aggression in male mice

Thiago S. Nakahara, Antonio P. Camargo, Pedro M. Magalhães, Mateus A. A. Souza, Pedro G. Ribeiro, Paulo H. Martins-Netto, Vinicius M. A. Carvalho, Juliana José, and Fabio Papes

Supplementary Figures:

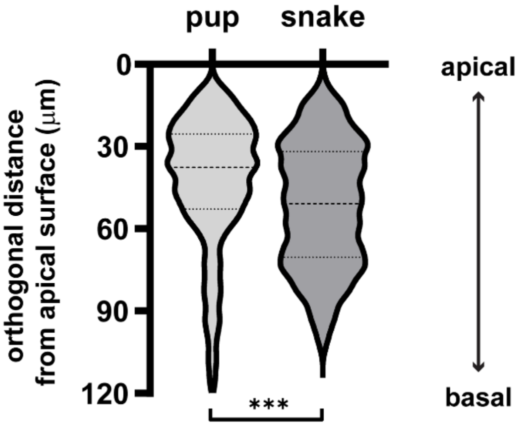

Supplementary Figure S1. VNO cells activated by pup odors are preferentially apically located in the sensory epithelium. Violin plots represent position of pS6-positive cells in the VNO epithelium in animals exposed to pup or snake odors, as shortest orthogonal distances from each stained cell to the apical (luminal) surface. N=350 pS6-positive cells for pup (from 4 sections; 40-95 stained cells per section) and N=819 pS6-positive cells for snake (from 6 sections; 28-225 stained cells per section). Dashed line indicates median; dotted lines represents interquartile range. Note the distribution of pS6-positive cells in both the apical and basal VNO layers for the snake odor, but comparatively distinct distribution of stained cells for pup odors, which are more apically located. ***P=0.001; two-sample Kolmogorov-Smirnov cumulative distribution test (D metric = 0.6875).

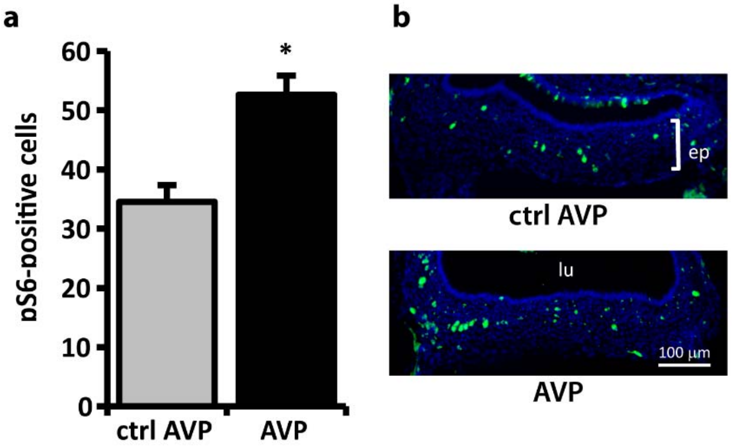

Supplementary Figure S2. Vasopressin administration increases pup-induced VNO activity in virgin male animals. **a,** Number of pS6-positive VNO cells (normalized to 50,000 μm^2 of sensory epithelium) in animals injected i.p. with vasopressin (AVP) on days 1, 3 and 5. Control (ctrl AVP) are animals injected with HBSS saline (vehicle). Exposure to pup odors was performed on day 5 (see Methods for details). n=3 animals; 6-9 sections per animal. *P<0.05, one-tailed Welch's t-test. See Supplementary Table S1 for statistical test metrics. **b,** Representative images of VNO coronal sections subjected to pS6 immunostaining (green fluorescence) for groups shown in **a**. DAPI nuclear stain is shown in blue. ep, VNO epithelium; lu, VNO lumen. Bar = 100 μm.

Supplementary Tables:

Files supplied separately as Excel spreadsheets

Supplementary Table Legends:

Supplementary Table S1. Detailed statistical values and metrics. The first tab exhibits means and standard errors of the means per individual, sample size (n is the number of animals), statistical tests and metrics, and P values. The second tab includes corresponding values calculated per section (sample size n is the total number of sections across animals).

Supplementary Table S2. Comprehensive list of transcripts coding for rhodopsin-like GPCRs annotated in the mouse genome. The list includes transcript isoform ID, gene acronyms, expression metric in male and female VNO RNA sequencing libraries. Quantification metric is mean TPM (transcripts per million reads), n=3 individuals for each sex.

Supplementary Table S3. List of genes from the transcripts in Supplementary Table S2 with FPKM expression above 0.5. The list includes transcript gene symbol, mean expression metric in VNO RNA sequencing libraries (n=3 males and 3 females) and annotation in the mouse genome (release 68).
